# Smart Release Nano-formulation of Cytochrome C and Hyaluronic Acid Induces Apoptosis in Cancer Cells

**DOI:** 10.4172/2157-7439.1000427

**Published:** 2017-02-24

**Authors:** CM Figueroa, BN Suárez, AM Molina, JC Fernández, Z Torres, K Griebenow

**Affiliations:** Department of Chemistry, University of Puerto Rico, Río Piedras Campus, San Juan, PR 00931, Puerto Rico

**Keywords:** Active targeting, Cancer therapy, Cytochrome c, Drug delivery, Enhanced-permeation retention effect, Hyaluronic acid, Nanoparticles, Smart release

## Abstract

Herein we tested a nanosized cancer-cell targeted delivery system based on cytochrome c (Cyt c) and hyaluronic acid. Cyt c was chosen since it is a *per se* non-toxic protein but causes apoptosis when delivered to the cytoplasm of target cells. Hyaluronic acid was employed to create the nanosized delivery system with passive targeting capability in order to exploit the enhanced permeation and retention (EPR) effect and active targeting capability of hyaluronic acid. In addition, our goal was to incorporate a smart release strategy to only promote protein release upon reaching its target.

Nanoparticles were formed by a simple yet precise nanoprecipitation process based on desolvation. They were physically characterized to select precipitation conditions leading to adequate size, shape, protein bioactivity, and protein loading to produce a feasible targeted cancer treatment. We synthesized nanoparticles of around 500 nm diameter with a 60% protein loading and more than 80% of protein bioactivity. *In vitro,* cumulative release of 92% of Cyt c was observed after 8 h under conditions mimicking the reductive intracellular environment, while under non-denaturing conditions only 20% was released. The nanoparticles displayed a selective cytotoxic effect on cancer cells. After 6 h of incubation with the nanoparticles, hyaluronic acid receptor over expressing A549 human lung adenocarcinoma cells showed a viability of ca. 20% at 0.16 mg/ml of Cyt c concentration. Only a negligible effect was observed on viability of COS-7 African green monkey kidney fibroblast, a normal cell line notoverexpressing the hyaluronic acid receptor. Confocal microscopy confirmed that the drug delivery system indeed delivered Cyt c to the cytoplasm of the target cells. We conclude that we were able to create a smart stimuli-responsive targeted drug delivery system with significant potential in cancer therapy.

## Graphical Abstract

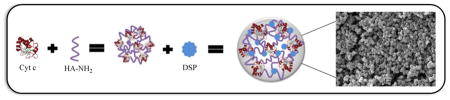

## Introduction

Cancer is a descriptive term for a cluster of heterogeneous diseases with some similarities according to the National Cancer Institute. Thus, there is no single therapeutic approach to eradicate the disease but for each type of cancer a specific approach has to be found. Nevertheless, there are common hallmarks of cancers that can be exploited to develop delivery and treatment strategies. For example, it is known that passive accumulation of nanoparticles (NPs) at solid tumors can be achieved by exploiting the Enhanced Permeation and Retention (EPR) effect, first described by Maeda and colleagues [1]. Tumors must generate blood vessels quite early to allow for sufficient nutrients to reach the core but the rapid growth of new vasculature produces leaky blood vessels and poor lymphatic drainage, which can be advantageously exploited in drug delivery systems (DDS) [2].

Designing nanoparticles for drug delivery has to be done keeping in mind that small particles (<10 nm) are excreted by the kidneys and large particles (>500 nm) are rapidly recognized and trapped by the reticuloendothelial system (RES) [3,4]. It has been found that DDS must accumulate through passive targeting in the tumor site first in order to take advantage of active targeting strategies if incorporated in the system [5]. Currently, some antibody-based therapeutics, i.e., Zevalin and Bexxar [6], are clinically approved as active targeting agents. Besides antibodies, active targeting can also be achieved by using ligands, e.g., transferrin, folate, integrin, and hyaluronic acid [7,8]. NP formation and stimuli responsive characteristics can be incorporated into DDS to improve targetability, therefore minimizing undesired side effects, which are one the main flaws of conventional cancer chemotherapy.

Besides the targetability of a DDS, another crucial point when constructing a DDS is the cytotoxic drug selection. Current chemotherapeutics, i.e., cisplatin, doxorubicin (DOX), affect the patients’ DNA [9] and hence there is a significant interest in the use of therapeutic proteins leaving no long-lasting therapeutic footprint [10]. Proteins are very specific and potent at their biological function and they can be relatively easily engineered for pharmaceutical purposes [11]. Another important advantage of therapeutic proteins in cancer therapy is their relative safety. Proteins are biodegradable and non-mutagenic biomacromolecules that will not disrupt or alter the patients’ DNA [12].

In this study we selected cytochrome c (Cyt c) as the model protein. Cyt c is a dual-function protein that induces programmed cell death once is released from the mitochondria upon recognition and response to apoptotic stimuli by the cell. These signals are ignored by cancer cells therefore the metabolic caspase cascade that must take place to induce apoptosis does not get activated [13]. It has been shown that once Cyt c is delivered directly into the cytoplasm of cancer cells it still induces apoptosis [14,15].

Protein formulation and NP formation requires extensive optimization work as each protein has distinctive physicochemical properties, such as, molecular weight and isoelectric point, and consequently synthesis conditions will vary greatly among proteins [16]. Proteins can be nano-precipitated by using solvent displacement methods, which were first developed by Fessi and colleagues [17]. However, a major disadvantage of their method is that it is mostly suitable for hydrophobic compounds and it is inefficient for many proteins since they are usually hydrophilic. Consequently, the technique was adjusted for proteins by employing water-miscible organic solvents to synthesize NPs [16]. That work showed that the selection of the adequate solvents is essential to enable nanoprecipitation of hydrophilic proteins. Despite the use of DMSO, a known protein-denaturing solvent [18], they were able to encapsulate lysozyme into polymeric NPs. Lysozyme is one of the most stable proteins and refolds into the native state from unfolded states and thus is not very representative of the usually quite labile pharmaceutical proteins for which unfolding is frequently irreversible. Subsequently, effects of the pH, the rate of addition of the desolvating agent, and the protein concentration on NP formation and stability have been characterized in detail [19,20]. The evolved technique has been proven successful to form small protein NPs than can be modified or loaded with diagnostics and/or therapeutic drugs such as DOX [21]. One of the advantages of using NPs to deliver therapeutics is that their size, shape, and surface charge can be controlled to incorporate passive, active, or stimuli-responsive targeting [4].

Polymers and glycans, such as, poly(ethylene glycol) and glycolic acid, have been used to modify proteins to overcome their low stability, immunogenicity, and short half-life [22,23]. Hyaluronic acid (HA) has been previously used in this manner to protect proteins from degradation [24,25] but in addition also as active ligand to target cancer cells since many of them overexpress the HA receptor, cluster-differentiation 44 (CD44) [26]. In a previous work, we developed a Cyt c-HA based DDS which showed systemic selectivity towards CD44-positive cancer cells when compared to CD44-negative normal cells [27]. We also showed that HA can be effectively used simultaneously as active ligand and as the integral building block of the DDS.

DDS can be engineered to behave in a predictable and controlled manner thus making them a “smart” DDS. This type of system takes advantage of either external or internal stimuli, such as light, magnetic field, pH, and redox activity, to trigger drug release and thus minimize side effects and maximize treatment efficacy [28,29]. Crosslinkers can easily be incorporated into the surface of NPs and disrupted once a particular stimulus is received. For example, the disulfide bonds that some cross linkers possess, are cleaved in the reductive intracellular environment where glutathione (GSH) represents the major reducing species [30].

In this work, we designed a one-step nanoprecipitation method to construct Cyt c and HA NPs. We incorporated a crosslinker containing a disulfide bond into the NPs and verified that it is solely cleaved under intracellular conditions subsequently resulting in the release of Cyt c. This system is not only passively and actively targeting cancer cells but it is furthermore a smart stimulus-responsive system that releases the apoptotic drug only once inside the cells. We tested various organic solvents in which both Cyt c and HA are insoluble so that NPs form when water is displaced upon solvent addition. Physical parameters were determined for each sample, and also, cytotoxicity and cellular uptake were evaluated.

## Materials and Methods

Cyt c from equine heart was from Sigma-Aldrich^®^ (St. Louis, MO) and HA-NH_2_ (10 kDa) from Creative PEGWorks (Winston-Salem, NC). The dithiobis(succinimidyl propionate) (DSP) cross linker was obtained from ProteoChem™ (Loves Park, IL). The cell lines A549 (human lung adenocarcinoma; ATCC^®^ CCL185 ™) and COS-7 (African green monkey kidney fibroblast) cells were from the American Type Culture Collection (Manassas, VA). CellTiter 96 aqueous non-radioactive cell proliferation assay was from Promega Corporation (Madison, WI). NucBlue^®^ Fixed Cell ReadyProbes^®^ Reagent (4′,6-diamidino-2-phenylindole, dihydrochloride, DAPI), fluorescein (FITC), Annexin Dead Cell Apoptosis Kit with Annexin V Alexa Fluor^®^ 488 and propidium iodide (PI) were purchased from Invitrogen (Carlsbad, CA). Caspase colorimetric assay kit was purchased from BioVision, Inc., San Francisco, CA and the caspase 9 substrate was from Sigma Aldrich (St. Louis, MO). All other chemicals (reagentor analytical grade) were purchased from various suppliers and used without further purification.

### Synthesis of Cyt c-HA NPs

**Figure 1:**
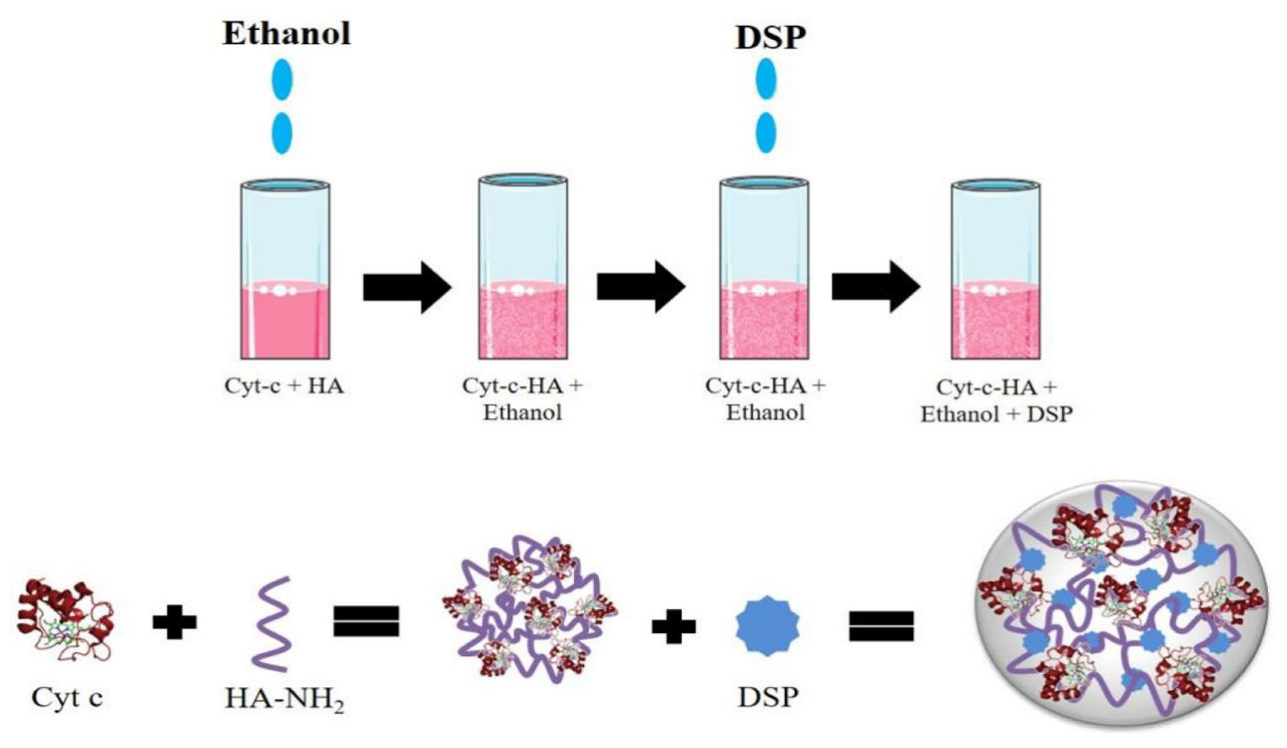
Scheme of the nano-formulation process of Cyt c-HA NPs.

Cyt c-HA NPs were formed through chemical modification and nanoprecipitation in principle following a published procedure with adaptions [31]. Optimization of the NP synthesis was achieved by changing several precipitation parameters and consequently, evaluating their effect on the physical properties of the NPs. In brief, native Cyt c and HA-NH_2_ (10 kDa or 1 MDa) were dissolved separately in nanopure water. Cyt c was then added drop-wise to the dissolved HA-NH_2_ testing various concentrations of each component. The solution was left stirring at a constant rate for 15 min and a 10 μl aliquot was taken for absorbance measurements at 408 nm (Cyt c heme group) in order to calculate physical parameters of the samples, e. g., drug loading. Self-assembled Cyt c-HA NPs were produced by desolvating the samples by adding a suitable organic solvent (ethanol or acetonitrile) with a syringe needle at a constant flow rate of 120 ml/h to achieve a final volume ratio of 1:4 water:solvent. The NPs suspension was left stirring for 15 min. Subsequently, different amounts of DSP were added to assess effects of the crosslinker concentration on the NP formation. After DSP addition, the Cyt c-HA NPs were stirred for 30 min followed by brief sonication and then they were centrifuged at 8000 rpm for 10 min at 4°C. Pellets were washed with nanopure water twice and freeze-dried. Table 1 summarizes selected conditions tested during NPs synthesis. Figure 1 shows a scheme of the NPs formation.

### NP physical characterization

#### NP yield, drug loading, and encapsulation efficiency

To calculate the NP yield, drug loading, and encapsulation efficiency, an aliquot of 10 μl was collected right before nanoprecipitation to determine the initial amount of Cyt c. The absorbance was measured at 408 nm (ε=6.7 ml/mg·cm^-1^). The final amount of NP was obtained by weighing the final product. To determine the amount of Cyt c in the NP, we resuspended a small known amount of each sample in 0.1 M PBS, 150 mM NaCl at pH 7.4 followed by sonication for 5 min to measure their scattering absorption spectra. Since α-lactalbumin does not have an absorption peak at 408 nm like Cyt c, we used α-lactalbumin-HA NPs scattering spectrum to subtract it from the Cyt c-HA NPs spectrum. From the subtraction, we calculated the Cyt c amount in the NPs. All measurements were done in triplicate. Equations (1) to (3) were used to calculate each parameter [32]:

(1) $$\text{Drug}\;\text{loading}\;\%=\left(\frac{\text{Weight}\;\text{of}\;\text{Cyt}\;c\;\text{in}\;\text{NPs}}{\text{Weight}\;\text{of}\;\text{NPs}}\right)\times 100$$

(2) $$\text{Nanoparticle}\;\text{yield}\;\%=\left(\frac{\text{Weight}\;\text{of}\;\text{NPs}}{\text{Weight}\;\text{of}\;\text{HA}+\text{initial}\;\text{drug}+\text{crosslinker}}\right)\times 100$$

(3) $$\text{Encapsulation}\;\text{efficiency}\;\%=\left(\frac{\text{Weight}\;\text{of}\;\text{Cyt}\;c\;\text{in}\;\text{NPs}}{\text{Weight}\;\text{of}\;\text{initial}\;\text{drug}}\right)\times 100$$

#### Scanning electron microscopy

The morphology of the NPs was observed through a JEOL 5800LV scanning electron microscope at 20 kV. Each sample was coated with gold for 15 sec using a Denton Vacuum DV-502A.

#### Dynamic light scattering

We used the Zetasizer Nanoseries from Malvern to determine the NPs’ size, polydispersity index, and zeta potential value for each sample. Samples were dissolved in water and placed in polystyrene disposable cuvettes for size measurements and folded capillary disposable cuvettes for zeta potential measurements. Each value is the average of three runs of around 15 measurements each.

#### CD spectroscopy

To obtain CD spectra of the samples and evaluate irreversible procedure-induced changes in the Cyt c structure, we nanoprecipitated Cyt c and HA as mentioned before but without adding the crosslinker. Samples were centrifuged and placed in a desiccator overnight to remove the organic solvent. The NPs were then re-dissolved in water in order to measure the absorbance of the loaded Cyt c. We used a JASCO J-1500 High Performance CD spectrometer at room temperature to obtain the spectra of the samples placed in 10 mm quartz cuvettes. Changes in the protein tertiary structure were observed at 260–350 nm and changes in the Soret band (heme group) were observed at 380–450 nm. The nanopure water blank was subtracted from each sample that was scanned thrice to obtain an averaged spectrum.

#### *In vitro* Cyt c release

To investigate release from our system, zero GSH was used as control, 1 μM GSH to simulate extracellular conditions, and 10 mM to simulate intracellular conditions [30]. GSH solutions were prepared fresh using the release buffer consisting of 50 mM PBS, 1 mM EDTA at pH 7.4. One ml of each of these three solutions was added to approximately 0.5 mg of each sample. Samples were left stirring at 37°C for various amounts of time. For measurement they were centrifuged for 10 min at 10,000 rpm and the supernatant was removed with a syringe to avoid pellet disruption. The supernatant absorbance was measured at 408 nm. Then, each sample was resuspended in fresh solution to maintain sink conditions. Release is presented as cumulative release. Release was measured in triplicate, the results averaged and the standard deviation calculated.

#### Cell-free caspase assay

Caspase activation by Cyt c was measured in A459 cell lysate following the procedure previously established in ref. [27]. Briefly, cells were grown as described in the cell culture method section. Once 85% confluency was reached, cells were centrifuged at 2,000 rpm for 5 min at 4°C and washed with fresh medium, then resuspended in cold PBS concentrating the pellets in the least amount of volume possible. After the final centrifugation, the pellet was resuspended in lysis buffer and cells were lysed with three freeze-and-thaw cycles using liquid nitrogen and a water bath at 37°C. The protein content in the supernatant (lysate) was determined using Coomassie Blue (595 nm) after centrifugation at 10,000 rpm for 20 min at 4°C. Lysate, samples, 50 μl of 2× reaction buffer, and 5 μl of caspase 9 substrate were added to a 96-well plate and incubated for 1 h at 37°C. The absorbance was measured at 410 nm and the average absorbance of lysate was used as blank and subtracted from all samples. Since Cyt c and the resultant product in the assay absorb around 410 nm we added a control of Cyt c without adding the caspase 9 substrate so its absorbance could be subtracted from those samples with the protein (Cyt c-HA NPs and Cyt c-HA NPs −DSP). This procedure was not necessary for HA and albumin (non-apoptotic protein, negative control) since they do not have a significant absorbance at this wavelength. Relative caspase activation was obtained using native Cyt c as control of 100% caspase activation. Samples were done in triplicate.

### Cell culture experiments

Two cell lines were grown to evaluate the DDS effect on cancer (A549) and normal (COS-7) cells. We selected COS-7 cells as negative control because it is a normal cell line not overexpressing CD44 [33] while A549 overexpresses CD44. A549 and COS-7 cells were cultured in Dulbecco’s Modified Eagle’s Medium (DMEM) containing 1% L-glutamine, 10% fetal bovine serum (FBS), and 1% penicillin. Cells were kept in a humidified incubator under 5% CO_2_ and 95% air at 37°C. Experiments were conducted before cells reached 25 passages. For cell viability experiments cells were seeded in 96-well plates for 24 h as for confocal microscopy but in chambered cover-slides (4 wells). Cell growth was arrested by decreasing the FBS concentration in the medium to 1% for 18 h. Then, cells were incubated with Cyt c-HA NPs and controls (native Cyt c and HA) for 6 h.

#### Cell viability

A549 cells were incubated for 6 h with various concentrations of Cyt c-HA NPs (0.022, 0.044, 0.089, and 0.164 mg/ml of loaded Cyt c) and controls (native Cyt c and HA-NH_2_). Controls and the sample at the highest Cyt c concentration tested in A549 cells were also incubated in COS-7 cells as negative controls. After sample incubation, we added 20 μl the MTS reagent from the CellTiter 96 aqueous non-radioactive cell proliferation assay to each well. The plate was placed at 37°C and 5% CO_2_ atmosphere for 1 h. Absorbance was measured at 492 nm using a microplate reader (Mean ± SD, n=8).

#### Apoptosis and NP uptake visualization

Confocal microscopy was used to visualize and confirm early and late apoptosis induction after incubation with NPs. We used the DAPI/PI/Annexin V staining in A549 cells as described in the protocol by Life Technologies to confirm apoptosis-mediated cell death. In brief, cells were seeded in a 4-chambered slide system and incubated with controls (native Cyt c and HA-NH_2_) and Cyt c-HA NPs at a 0.06 mg/ml Cyt c concentration at 37°C for 24 h. Cells were washed with PBS and incubated with Annexin and PI for five minutes. After the probe incubation, cells were washed with PBS thrice and fixated with 3.7% formaldehyde for 30 min followed by washes with PBS. DAPI was added to each well after fixation followed by three PBS washing cycles. To avoid photobleaching of the fluorescent dyes, we added 200 μl of glycerol to each well. The slides were then covered with coverslips. If slides were not observed immediately they were left overnight at room temperature and then at 4°C until visualization under the microscope. After incubation, the chambered plates were observed under a Nikon Eclipse Ti microscope, using a 40× oil objective to assess co-localization of DAPI, Annexin V, and PI in cells. While DAPI stains the cellular nucleus blue, Annexin V (shows green fluorescence because is bound to FITC) stains apoptotic cells and PI dead or necrotic cells. Annexin V detects phosphatidyl serine on the cell membrane that has been translocated to the outside of the cells which is a sign of early apoptosis while PI stains DNA in the nucleus only when there has been membrane permeabilization. The fluorescent probes were excited at the following wavelength: DAPI=402 nm, Annexin V=487 nm, and PI=561 nm. The emission of DAPI was observed at 420–480 nm, of Annexin V at 525 nm, and of PI at 595 nm.

To observe cellular internalization, samples were labeled with FITC. Controls (HA and Cyt c) and Cyt c-HA NPs were reacted with FITC. To link FITC to the respective samples, FITC was dissolved in DMSO to a final concentration of 1 mg/ml. Next, 50 μl was added to 1 ml of each sample dissolved in PBS buffer. The reaction was stirred for 3 h in the dark at 4°C. Samples were centrifuged and lyophilized. A549 and COS-7 cells were incubated with HA-NH_2_-FITC, Cyt c-FITC, and NPs (Cyt c concentration of 0.06 mg/ml) at 37°C for 6 h to evaluate specificity to cancer cells. After removing the media, chambers were washed thrice with PBS following fixation with formaldehyde and washing with PBS. After fixation, DAPI was added to observe the cellular nuclei. We used glycerol to avoid photobleaching. Plates were left overnight at room temperature and observed by confocal microscopy using the microscope mentioned before with a 20× objective. FITC was excited at 487 nm and observed at 525 nm.

### Statistical analysis

All experiments were done at least in triplicate unless stated otherwise. Values are the means ± SD. The Mann-Whitney U Rank Sum test was completed using the Sigma Plot 11.0 software to determine statistically significant differences (p<0.05) between Cyt c-HA NPs in cell viability experiments and controls (Cyt c and or HA-NH_2_).

## Results and Discussion

### NP formation

**Figure 2:**
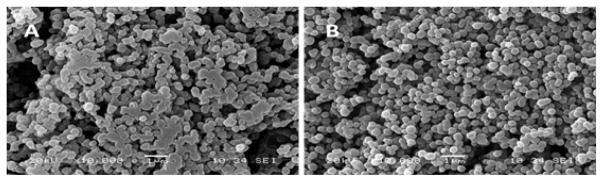
SEM images of Cyt c-HA NPs synthesized at two crosslinker concentration, either 3 mg/ml (A) or 5 mg/ml (B).

This project had several intermediate goals to finally accomplish the construction of a “smart” multifunctional DDS. The first one was to obtain nano-sized particles that fulfill the requirements to enable tumor accumulation by the EPR effect. Second, we aimed at maintaining Cyt c bioactivity to induce apoptosis after NP formation. Third, we aimed at reducing the incubation time required to induce cytotoxicity in cancer cells compared to our previous DDS [27]. Fourth, we expected to produce a smart release stimuli-responsive DDS. Since obtaining nanosized particles was a significant issue, samples were observed by SEM right after formation. NPs were formed using a one-step protein nanoprecipitation method followed by crosslinking Cyt c and HA to avoid dissolution in the aqueous phase. Conditions were varied until achieving smooth and spherical NPs (Figure 2). When the observed NPs shape and size were reasonably within our goal, we proceeded to confirm it by DLS. This step proved to be challenging since the NPs’ size increment due to the hydrodynamic radius in solution could be relatively high. For example, NPs in SEM could have a diameter of around 150–200 nm and in DLS of around 800 nm. This change was significantly higher when NPs were synthesized using a high molecular weight HA-NH_2_ (1 MDa). To take advantage of the EPR effect, NPs need to be smaller than 800 nm [34], so the hydrodynamic radius was a big concern. To address this issue, we selected a smaller HA-NH_2_ (10 kDa) to obtain smaller NPs in solution. This difference in size among this two techniques can be due to the water retention ability of HA. It is known that HA can retain high amounts of water [35] therefore it might produce a higher NP size when observed dissolved by DLS instead of under vacuum in the SEM (Table 1). NPs suspension is a critical step when working with DDS. We used sonication after NP formation to produce a proper suspension to avoid aggregation of the NPs.

According to the results, the best organic solvent in the nanoprecipitation process was ethanol. Also, the optimum protein to polymer ratio, 5:2, was essential to produce the desired NPs. Although many more conditions were tested, two conditions were selected to carry out further experiments: Cyt c-HA NPs, with a crosslinker concentration of 5 mg/ml, and Cyt c-HA NPs (3) (3 representing the crosslinker concentration, 3 mg/ml).

### Physical characterization of NP

#### Dynamic light scattering

The selected samples were physically characterized as mentioned above. The size and zeta potential values were measured by DLS. Sample Cyt c-HA NPs (3) had a larger diameter (542 ± 9 nm) and polydispersity index (0.202 ± 0.03) than Cyt c-HA NPs (504 ± 7 nm and 0.057 ± 0.04, respectively). A low polydispersity index (PDI) indicates a narrow NP size distribution, which is preferable for a DDS as well as small diameter. The zeta potential of Cyt c-HA NPs (3) was slightly smaller (-28.7±0.6 mV) than that of Cyt c-HA NPs (−32.0±0.2 mV). These values represent the magnitude of the electrostatic forces around the NP, which can repulse or attract neighboring particles and produce flocculation by NP aggregation or a stable suspension. The zeta potential is also related to the particle stability. Samples with zeta potentials higher than 30 mV and −30 mV are considered to be stable [36]. Therefore, Cyt c-HA NPs are slightly more stable than sample Cyt c-HA NPs (3). This was clearly observed with the naked eye, Cyt c-HA NPs in water, buffer, or media remained stabile in suspension while Cyt c-HA NPs (3) flocculated over time. To continue their physical characterization, parameters from equations 1 through 3 were then calculated for these two samples.

#### NP yield, drug loading, and encapsulation efficiency

**Figure 3:**
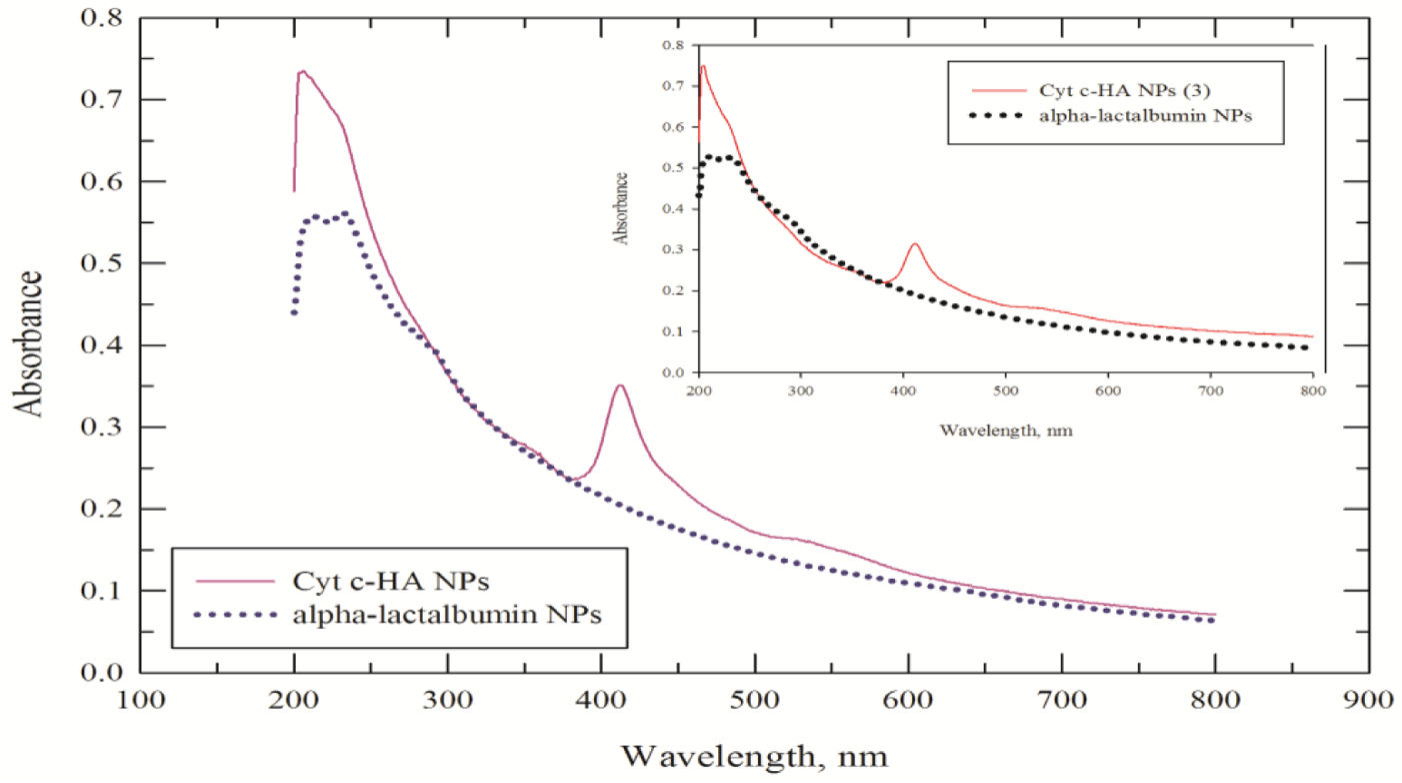
Scattering spectra of Cyt c-HA NPs (purple line), Cyt c-HA NPs (3) (red line) and α-lactalbumin NPs (blue and black dotted lines).

Figure 3 shows the Cyt c-HA NPs, Cyt c-HA NPs (3), and α-lactalbumin NPs scattering spectra. The latter was subtracted to obtain the residual absorbance at 408 nm and calculate the amount of Cyt c in the NPs. This value was used to calculate drug loading and encapsulation efficiency percentages for each sample (Table 1). In general, the nanoprecipitation yield, drug loading, and encapsulation efficiency percentages were higher for Cyt c-HA NPs than for Cyt c-HA NPs (3). Nanoprecipitation yield and drug loading percentages were both larger than 60% in Cyt c-HA NPs while encapsulation efficiency was lower (40 ± 2%). It is clear that the crosslinker amount has an important role in the formation of the NPs. The protein loss due to low crosslinker concentration was empirically observed during centrifugation after NP formation using 3 mg/ml crosslinker concentration (Cyt c-HA NPs (3)). The supernatant had an intense dark red color, characteristic of Cyt c, showing that a significant amount of the protein was not been encapsulated in the NPs. From this point forward we performed the following experiments only with the Cyt c-HA NPs sample.

#### CD spectroscopy

**Figure 4:**
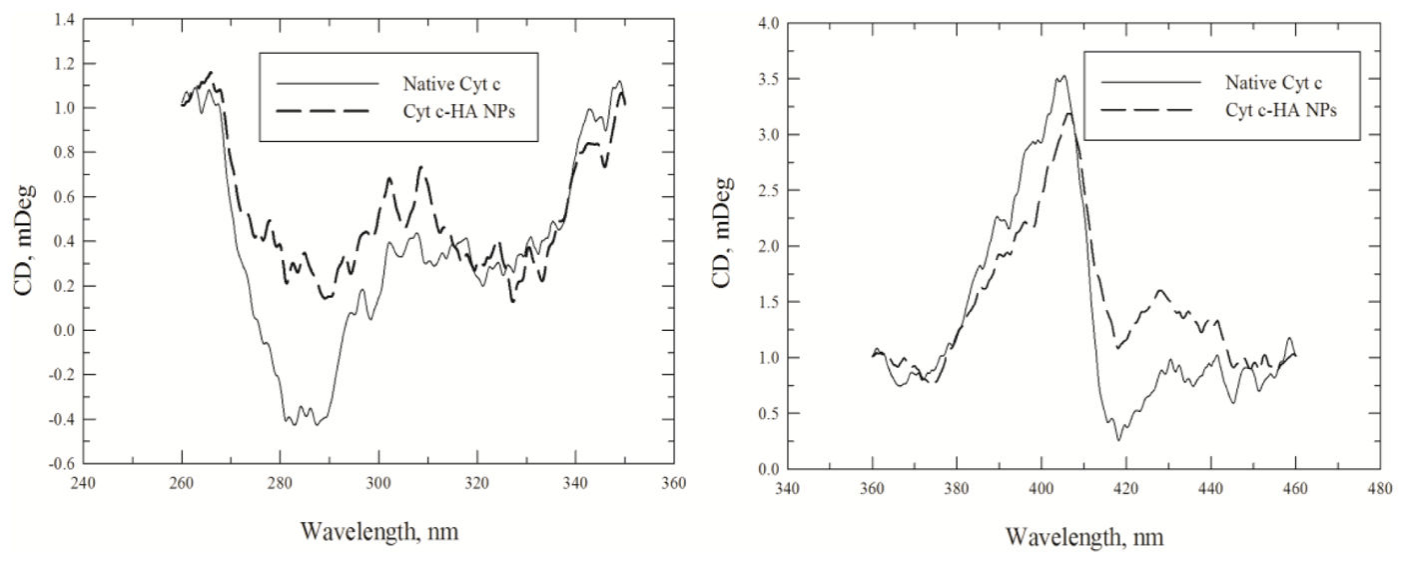
CD spectra of Cyt c-HA NPs in the near UV (left panel) and heme band region (right panel). Cyt c in the NPs (dashed line) was compared to native Cyt c (black line).

To obtain the CD spectra of loaded Cyt c, the NPs were formed without the crosslinking step. Note that the protein must be dissolved to obtain its CD spectrum because scattering leads to very low signal and thus noise in the spectra. Figure 4 shows the Cyt c CD spectra prior to and after NP formation. We observed moderate changes in the tertiary structure located in the near-UV region (260–340 nm, Figure 4 left panel) while relatively small changes were perceived in the Soret region (360–450 nm, Figure 4 right panel), characteristic of the Cyt c heme group’s environment. These changes in the tertiary structure can be produced by the nanoprecipitation process, the use of an organic solvent, and the physical purification process to synthesize the NPs. Even though the heme group appears to have little disruption from the synthesis of the NPs, we assessed Cyt c bioactivity to confirm its cytotoxic ability through a cell-free caspase activation assay.

#### Cumulative Cyt c release profile

**Figure 5:**
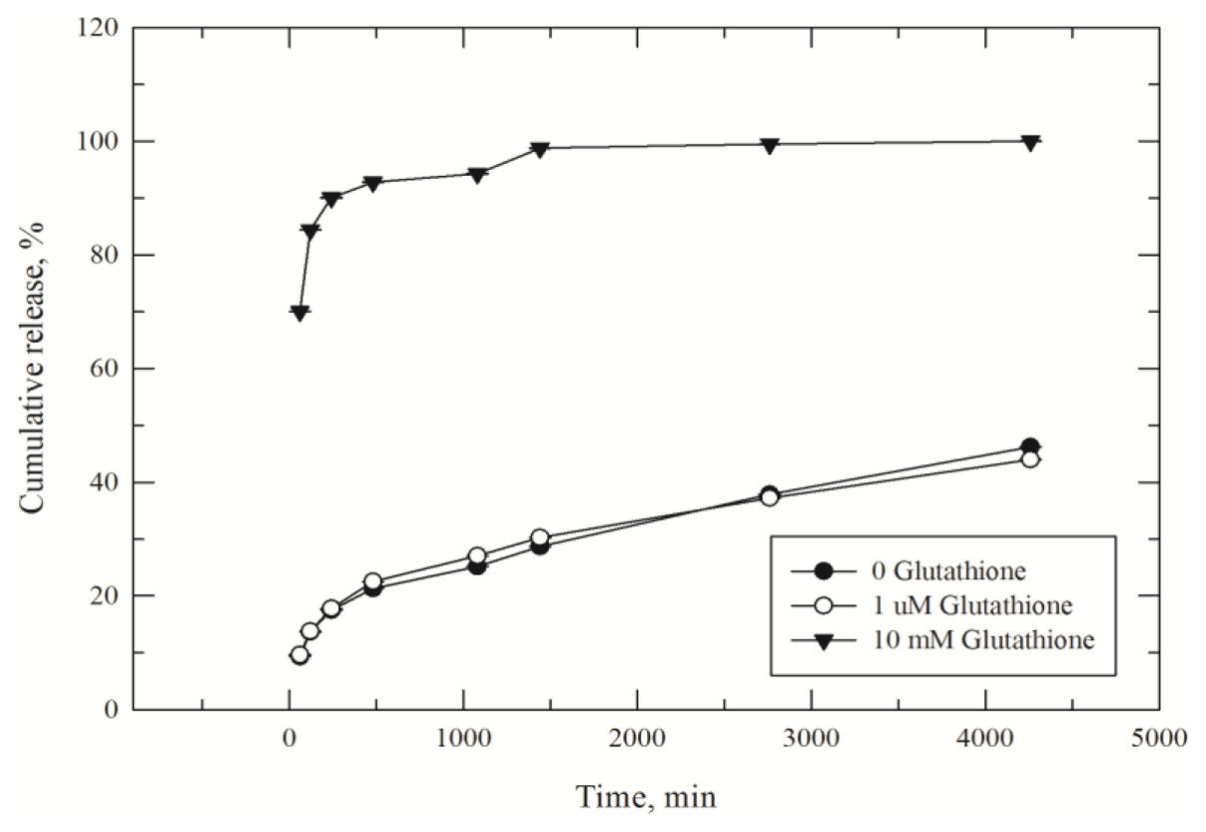
Cumulative *in vitro* release of Cyt c from the DDS. Cyt c-HA NPs were dissolved in PBS buffer with zero glutathione (black dots), 1 μM glutathione (white dots), and 10 mM glutathione (black triangles) to simulate extra- and intracellular physiological conditions. Data are the mean ± SD of three measurements.

To prove that we constructed a stimulus-responsive DDS, we tested extra- and intracellular conditions. The concentrations of GSH vary significantly outside of the cell (1 μM) and intracellularly (10 mM) which allows for the disruption of the disulfide bond present in the crosslinker only once inside the cells. A cumulative Cyt c release profile was obtained by incubating the NPs in 10 mM GSH. This system showed an initial burst in the first few hours followed by slow release of the remaining protein. It is important to emphasize that this type of release was only observed under intracellular conditions (10 mM GSH, 37°C, Figure 5). It reached near 100% Cyt c release around 24 h after incubation with GSH. At 6 h, where we measured cell viability, caspase activation, and cellular uptake, around 92% of the loaded Cyt c was released. Cyt c was not significantly released under extracellular conditions (1 μM GSH), which makes the DDS developed a “smart” stimulus-responsive release system.

### Cell culture experiments

#### Caspase activation

**Figure 6:**
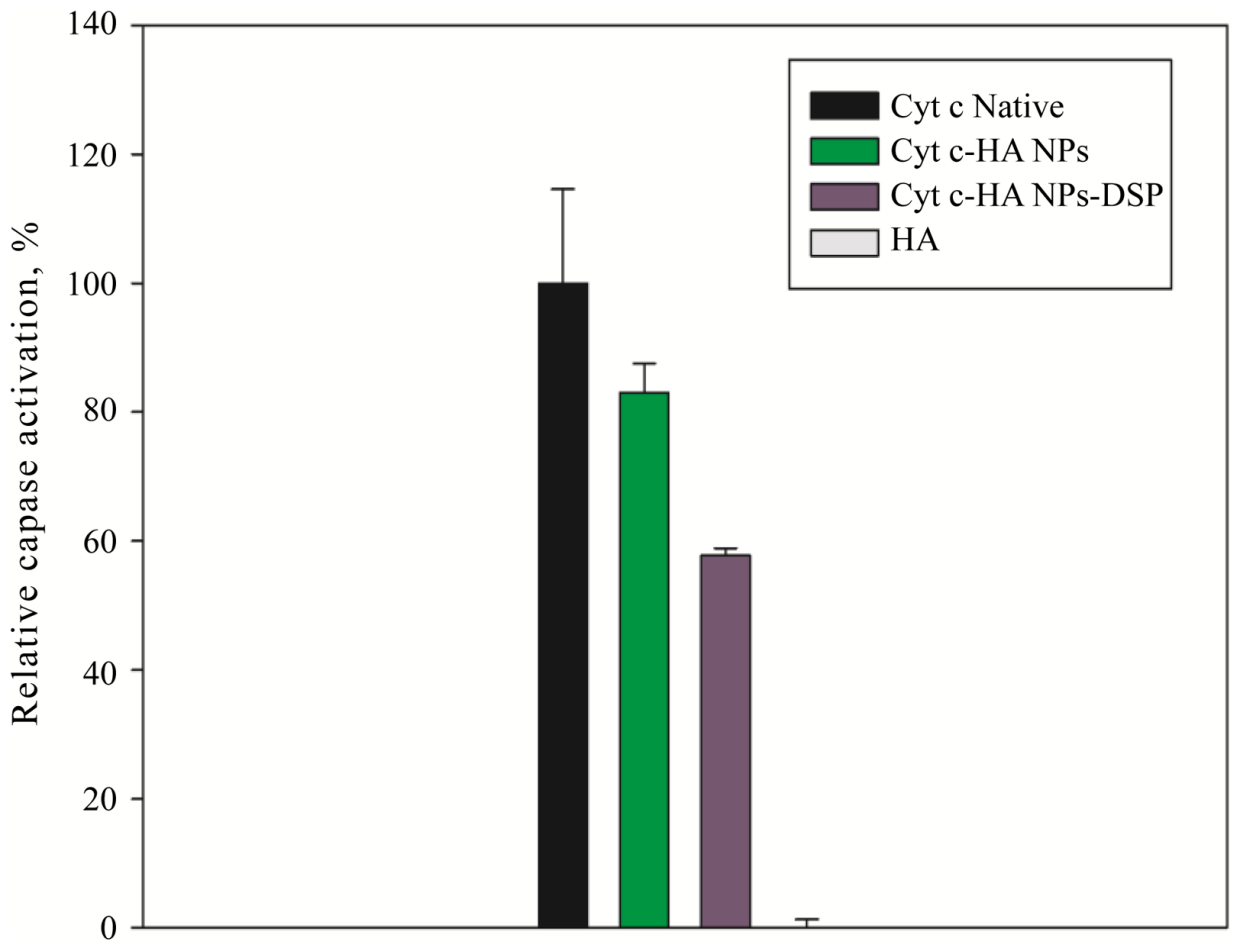
Relative caspase activation by Cyt c-HA NPs. Native Cyt c was used as positive control (black bar) while HA was used as negative control (gray bar, not observed). Cyt c-HA NPs with (green bar) and without (violet bar) crosslinker (DSP) showed significant caspase activation once released from the NPs.

As any protein, Cyt c can suffer procedure-induced structural changes as seen in our CD experiments, and bioactivity changes when used in synthetic procedures. To assess changes in the Cyt c capacity to induce cell death, we measured the relative caspase activation of Cyt c after NPs formation. Cyt c interacts with Apaf-1 in the cytoplasm to form the apoptosome and subsequently initiate the caspase cascade leading to programmed cell death [37]. With this assay, we evaluated the activation of the initiator caspase 9 to assess the ability of the system to activate upstream caspases. As seen in Figure 6, Cyt c-HA NPs can activate caspase 9 83 ± 5% relative to Native Cyt c (100%) while HA does not have any relative effect on its activation. Interestingly, once the crosslinker was not used, Cyt c decreased its caspase activation to 58 ± 1%. This can be due to a protective action afforded by the crosslinker when stabilizing the NPs. Considering that Cyt c was subjected to a relatively harsh procedure to form the NPs (agitation, desolvation with an organic solvent) it still significantly activated caspase 9.

#### Cell viability in A549 and COS-7 cells

**Figure 7:**
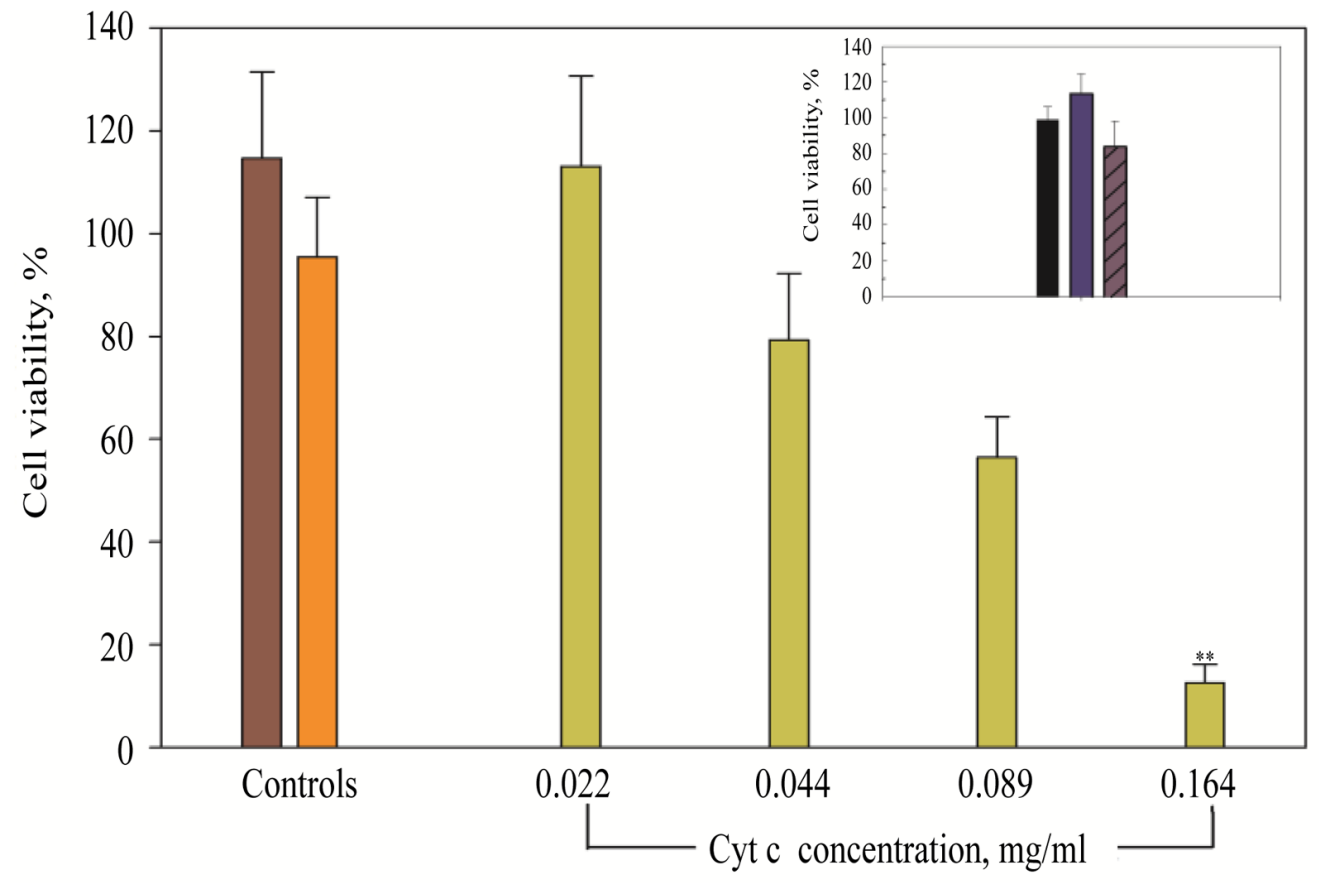
Dose response curve of Cyt c-HA NPs on A549 cells. Cyt c-HA NPs cytotoxicity was measured after 6 h of incubation in A549 cells (green bars) and COS-7 cells (insert). Negative controls were also tested in both cell lines: A549 cells (HA=brown bar, Native Cyt c=orange bar) and COS-7 cells (HA=black bar, Native Cyt c=blue bar, and Cyt c-HA NPs=purple with diagonal lines bar). Asterisks (**p<0.001) represent the significant statistical difference between Cyt c-HA NPs incubated in A549 cells and in COS-7 cells.

To investigate Cyt c’s ability to induce apoptosis, a dose response curve was constructed for A549 cells incubating them with 0.164, 0.088, 0.044, and 0.022 mg/ml of loaded Cyt c NPs for 6 h. Surprisingly, the incubation time was significantly reduced from 24 h to 6 h to observe a noteworthy reduction in cell viability as in our previous work [27]. The synthesized bioconjugates affected cell viability only after 24 h of sample incubation while with this improved smart release DDS the incubation time was reduced by 75% to observed a significantly lower cell viability. A clear relation between concentration and cell viability was observed (Figure 7) while controls did not affect cell viability (HA-NH_2_ and native Cyt c). The highest concentration of Cyt c tested reduced viability to 13 ± 4%. To confirm that the cytotoxicity was due to the Cyt c in the drug delivery system and not intrinsic Cyt c, 0.2 mg/ml of native Cyt c were added to the cells and indeed produced only a reduction to 95 ± 12% in cell viability. As seen in Figure 7 inset, neither Cyt c-HA NPs nor controls (HA-NH_2_ and native Cyt c) produced significant effects in COS-7 cell viability after sample incubation. These results show that we produced a targeted DDS that had a significant cytotoxic effect on cancer cells over xpressing CD44 and not on normal cells.

#### Apoptosis induction and DDS internalization

**Figure 8:**
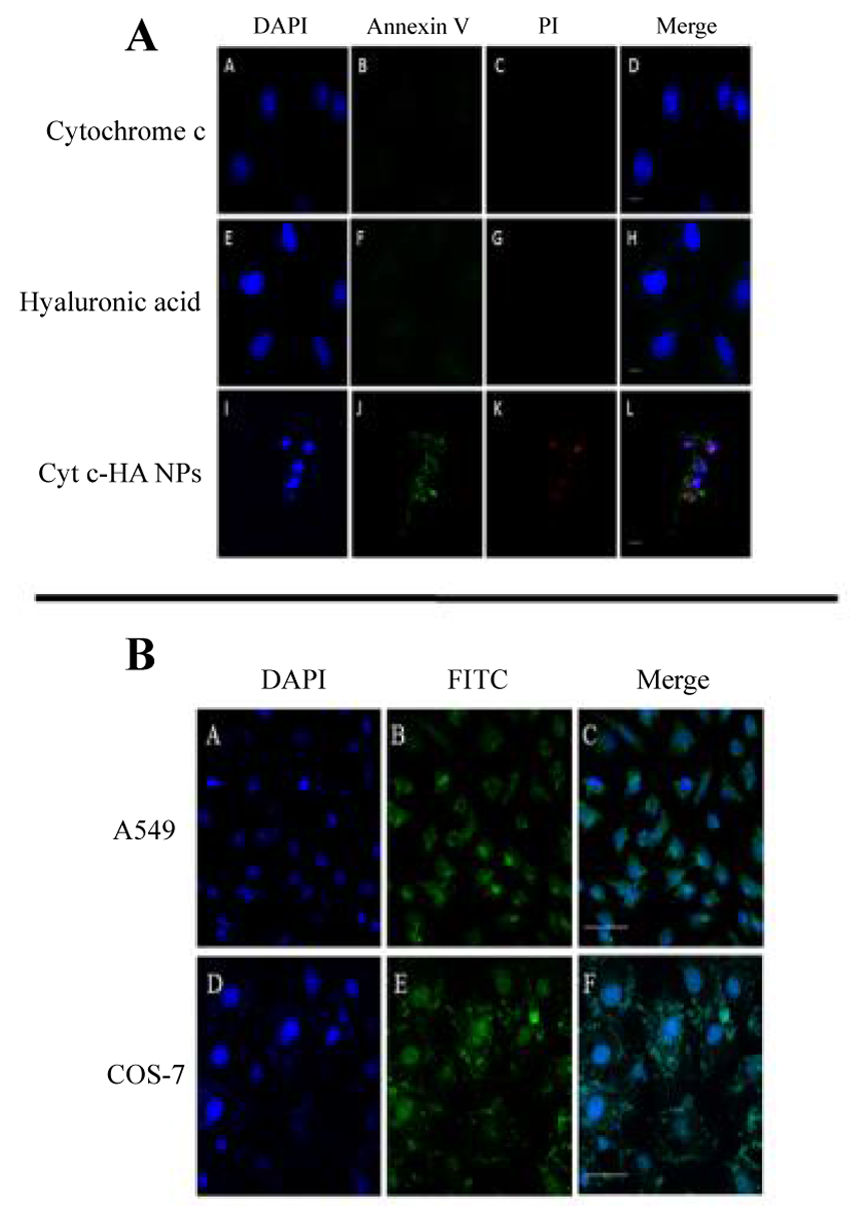
Confocal microscopy images after sample incubation. (A) Apoptosis induction after sample incubation in A549 cells. Cyt c (top row) shows no apoptosis induction similar to HA (middle row) while Cyt c-HA NPs show significant early (Annexin V=green dye) and late (PI=red dye) apoptosis induction. Cells were observed under a 40× objective. Bar scale represents 10 μm. (B) Cyt c-HA NPs-FITC cellular uptake visualized in A549 and COS-7 cells. Negative (COS-7) and positive (A549) CD44 cells were incubated with the NPs to observe preferential internalization. A549 cells (top row) show sample internalization (green fluorescence) while COS-7 cells (bottom row) display sample accumulation around the cells but no fluorescence inside the cell. DAPI (blue dye) was used to stain cell nuclei. Cells were observed under a 20× objective. Bar scale represents 50 μm.

Apoptosis induction was confirmed using confocal laser scanning microscopy. A549 cells were grown in chambered slides as mentioned before. The probes used in this experiment allow the detection of early apoptosis and dead or necrotic cells. When cells are undergoing apoptosis their cell membrane is disrupted exposing phosphatidylserine (PS) to the extracellular space. Annexin V is a fluorescent probe that can discriminate healthy and apoptotic cells since it binds PS only when it is exposed during the first stages of apoptosis. Late apoptosis or necrosis allow the access of fluorescent DNA-binding dyes, such as PI, to the disrupted and permeable nucleus, therefore, staining nucleic acids [38]. Cells in early apoptosis will only exhibit green fluorescence. However, in our case, both green and red fluorescence were detected which means cells underwent apoptosis-mediated cell death. This experiment confirmed apoptosis induction only when the drug delivery system was properly synthesized and delivered into the cells. Cyt c in its native form is not membrane permeable [39] therefore no apoptosis was observed in the cells (Figure 8A–8C, panel A). Incubation with HA-NH_2_ did not cause cytotoxicity either, as seen in Figure 8D–8F, panel A. In contrast, cytotoxicity was observed when exogenous Cyt c loaded into the NPs was delivered into the cytoplasm (Figure 8G–8I, panel A).

A cellular internalization experiment also confirmed the specificity of the DDS towards CD44 positive cancer cells. A549 and COS-7 cells were both incubated with Cyt c-HA NPs modified with FITC (0.05 mg/ml) for 6 h. Modified-FITC controls and NPs were incubated with cells to visualize specific cellular internalization of the system. In this case, Cyt c-HA NPs remained mainly accumulated extracellularly when incubated with COS-7 cells (CD44 negative normal cells) while they were clearly internalized in A549 cells (Figure 8, panel B).

## Conclusions

In this work, we describe the synthesis of a smart and multifunctional DDS. For the first time, we demonstrate synthesis of NPs comprised of Cyt c and HA. Our work was the first attempt to nanoprecipitate Cyt c and HA simultaneously to form a DDS with both, passive and active delivery, without the need for additional modifications. To further improve this system, we also incorporated a stimulus-response molecule that allows the stabilization of the NPs in aqueous solution and the Cyt c release in a controlled manner. The system was able to selectively induce cytotoxicity in CD44+ cancer cells after only 6 h of sample incubation which is a significant improvement from our previous DDS. This system has the potential to be an effective multifunctional smart-release DDS for cancer therapy.

## Figures and Tables

**Table 1:** Physical characterization of Cyt c-HA nanoparticles.

Sample	DSP, mg/ml	Nanoprecipitation yield, %	Drug loading, %	Encapsulation efficiency, %	Size, nm^	PDI*	Zeta potential, mV
Cyt c-HA NPs	3	47.5 ± 0.4	38.5 ± 0.6	18.9 ± 0.9	542 ± 9	0.202 ± 0.03	−28.7 ± 0.6
Cyt c-HA NPs	5	64 ± 4	61.6 ± 0.8	40 ± 2	504 ± 7	0.057 ± 0.04	−32.0 ± 0.2

^ size: diameter,

* PDI: Polydispersity Index
